# The membrane transporter lactose permease increases lipid bilayer bending rigidity

**DOI:** 10.1016/j.bpj.2021.06.038

**Published:** 2021-07-15

**Authors:** Nestor Lopez Mora, Heather E. Findlay, Nicholas J. Brooks, Sowmya Purushothaman, Oscar Ces, Paula J. Booth

**Affiliations:** 1 Department of Chemistry, Kings College London, London, United Kingdom; 2 Department of Chemistry, Imperial College London, London, United Kingdom; 3 Beyond Meat, El Segundo, California

## Abstract

Cellular life relies on membranes, which provide a resilient and adaptive cell boundary. Many essential processes depend upon the ease with which the membrane is able to deform and bend, features that can be characterized by the bending rigidity. Quantitative investigations of such mechanical properties of biological membranes have primarily been undertaken in solely lipid bilayers and frequently in the absence of buffers. In contrast, much less is known about the influence of integral membrane proteins on bending rigidity under physiological conditions. We focus on an exemplar member of the ubiquitous major facilitator superfamily of transporters and assess the influence of lactose permease on the bending rigidity of lipid bilayers. Fluctuation analysis of giant unilamellar vesicles (GUVs) is a useful means to measure bending rigidity. We find that using a hydrogel substrate produces GUVs that are well suited to fluctuation analysis. Moreover, the hydrogel method is amenable to both physiological salt concentrations and anionic lipids, which are important to mimic key aspects of the native lactose permease membrane. Varying the fraction of the anionic lipid in the lipid mixture DOPC/DOPE/DOPG allows us to assess the dependence of membrane bending rigidity on the topology and concentration of an integral membrane protein in the lipid bilayer of GUVs. The bending rigidity gradually increases with the incorporation of lactose permease, but there is no further increase with greater amounts of the protein in the membrane.

## Significance

Because of the relevance of protein-membrane interactions on cellular functions, in this work we address a fundamental question about the intrinsic role of proteins that are integral to the membrane itself on the ability of the membrane to bend. We use cellular-sized, giant vesicles (GUVs) that are commonly used in studies investigating cell membrane biophysics. However, we prepare protein GUVs by a novel, to our knowledge, method using hydrogel scaffolds, as this produces more robust, reproducible vesicles that can withstand physiological buffers than more generally used preparation protocols. We reconstitute a membrane protein, the lactose permease of *Escherichia coli* (LacY), into GUVs and assessed the influence of an integral membrane protein on the membrane rigidity under physiological conditions by fluctuation analysis.

## Introduction

Membranes are fundamental to cellular integrity and play active roles in many biological processes ranging from cellular signaling, transport and energy production to endocytosis and fusion (1,2). Cellular membranes must be mechanically robust as well as sufficiently malleable for membrane rearrangements that are inherent to cell growth and function, all while remaining intact. A fundamental physical property that underpins these essential cellular events is the capacity of the membrane to alter its curvature, with membrane bending rigidity being one of the main parameters that determines the energetics of curvature change (3, 4, 5, 6). Although studies have amassed on the influence of peripheral proteins that induce curvature in membranes, such as BAR domains (7), there has been less focus on the intrinsic role of proteins that are integral to the membrane itself (8) on this key ability of the membrane to bend.

Several different techniques have been used to determine bending rigidity, including methods that measure the force to actively bend a membrane (such as magnetic or optical traps (9,10) and micropipette aspiration (11, 12, 13)), as well as those based on the analysis of thermal fluctuations (14, 15, 16), x-ray scattering (17,18), or molecular dynamic simulations (19). The influence of membrane proteins on bending rigidity is unclear, and the few existing studies give differing results, partly because of the technique used and partly differing sample conditions (6), Micropipette aspiration has shown that a Ca^2+^ ATPase lowered membrane stiffness (20), whereas incorporation of bacteriorhodopsin had little effect on rigidity (21). Molecular dynamic simulations have suggested that the presence of membrane proteins at physiological amounts reduces membrane stiffness (22). In contrast, fluctuation analysis has shown that the presence of a single transmembrane helix increases bending rigidity (23).

Because methods such as micropipette aspiration tend to give lower bending rigidity values (24), we use fluctuation analysis to quantify bending rigidity. Experimental samples have centered on proteins and peptides reconstituted into giant unilamellar vesicles (GUVs). GUVs provide one of the simplest cell models, being of similar size, and thus overall curvature, as natural cells (25). GUVs made of amphiphilic phospholipids or polymers allow the control of the bilayer structure (26), membrane composition and asymmetry (27,28), encapsulation of bio-macromolecules (29), and cells (30). Despite those achievements, the functionalization of the lipid bilayer for the production of membrane-engineered GUVs with the ability to mimic structural and functional characteristics of biological cell membranes is still challenging and method dependent. Thus, the construction of engineered GUVs containing elements of native cellular membranes and without artifacts are highly valuable for their use in the determination of bending rigidity and the characterization of fundamental cellular features such as the protein-membrane interaction (31) and protein crowding (32).

Bending rigidity measurements require robust, defect-free GUVs that remain intact throughout fluctuation analysis. However, GUV samples vary according to their preparation method. Traditional GUV production methods have been used thus far for bending rigidity measurements. These methods, including gentle rehydration, give GUVs with a broad size distribution with high amounts of multilamellar vesicles. The widely used electroformation method requires judicious choice of electric field parameters to avoid lipid decomposition and works less well for physiological buffers and anionic lipids (33,34). Here, we exploit a new, to our knowledge, method. Hydrogels made of dextran polymers cross-linked by poly(ethylene glycol) (DexPEG) have proven to be a good alternative for the production of dense suspensions of GUVs with a controlled size distribution using different rehydration conditions and lipid classes (35). We make use of the hydrogel method to produce high-quality GUVs and compare their bending rigidity to GUVs prepared by electroformation. We also reconstitute lactose permease of *Escherichia coli* (LacY) into GUVs (giving LacY GUVs) in different lipid mixtures and buffers. We use a reconstitution method based on that of Bassereau et al. (16), which has been shown to give active membrane proteins in the GUV, although in this report we focus on establishing a method for accurate determination of bending rigidity when a membrane protein is present rather than assessing function. To this end, we exploit a fluorescently tagged LacY version to determine the actual protein concentration present in the GUV lipid bilayer, rather than rely on the protein/lipid ratio initially mixed together in LUVs, to determine the actual protein concentration in the GUV membrane for the bending rigidity measurements, i.e., postreconstitution. These protein concentrations were lower than that predicted from the initial protein concentration used for reconstitution in LUVs. This illustrates the unreliability of these initial values that are usually used to estimate the amount of finally reconstituted protein, as protein is lost during the reconstitution process. We find that less efficient reconstitution occurs at higher protein concentrations.

## Materials and methods

### General

Details of protein expression and purification (36), protein labeling (Fig. S1), and DexPEG hydrogel preparation (35) can be found in the Supporting materials and methods.

### LacY LUVs

Large unilamellar vesicles (LUVs) were formed by extrusion with the lipid mixtures 1,2-dioleoyl-glycero-3-phosphocholine (DOPC)/1,2-dioleoyl-glycero-3-phosphoethanolamine (DOPE) (50:50 mol%), DOPC/DOPE/1,2-dioleoyl-glycero-3phosphoglycerol (DOPG) (40:40:20 mol%), and DOPC/DOPE/DOPG (20:20:60 mol%) (Avanti Polar Lipids, Alabaster, AL). Briefly, stock solutions of the lipids in cyclohexane (50 mg/mL) were mixed to the desired lipid ratio. 200 *μ*L of the stock lipid mixture were dried under nitrogen gas and placed under vacuum overnight to form a lipid film. The lipid film was hydrated with 1 mL sodium phosphate buffer (50 mM (pH 7.4)), referred as NaPhos) and extruded 41 times with either 100 or 400 nm polycarbonate filters to obtain LUVs with a final lipid concentration of 10 mg/mL. Then, LUVs were incubated for 15 min with n-octyl-*β*-D-glucopyranoside (OG, 50 *μ*L, 610 mM; Anatrace, Maumee, OH) before protein insertion. LacY was overexpressed and purified in *E. coli* following a procedure published elsewhere (36). LacY was reconstituted into detergent-destabilized LUVs as previously described (37). Briefly, LacY purified and solubilized in n-dodecyl-*β*-D-maltopyranoside (DDM, 39 mM; Anatrace) was added to the previously OG-destabilized LUVs in the theoretically calculated protein/lipid molar ratios of 1:1 × 10^4^, 1:5 × 10^3^, and 1:2.5 × 10^3^. LacY was reconstituted for 45 min at room temperature and constant shaking to produce LacY LUVs. Optimized lipid compositions were selected based on their reconstitution efficiency and correct topology upon LacY insertion according to previous work (38). For instance, DOPC/DOPE (50:50 mol%) yields the correct LacY topology (∼72%) with acceptable reconstitution efficiencies (>75%), whereas the incorporation of the anionic lipid DOPG to the lipid composition increases the reconstitution efficiencies to 85%, but high amounts of DOPG proved detrimental for LacY topology, decreasing the correct topology to 20% with the lipid mixture DOPC/DOPE/DOPG (20:20:60 mol%). The excess detergent was removed with Bio-Beads SM2 (200 mg; Bio-Rad, Hercules, CA), in three sequential incubation steps of 1 h each. Additionally, LacY LUVs were dialyzed for 2 days or diluted in a volume of 23 mL NaPhos buffer (0.41 mg/mL lipids) and then centrifuged at 475,900 relative centrifugal force for 2 h to pellet LacY LUVs. Finally, LacY LUVs were redispersed in 500 *μ*L NaPhos buffer to give a final lipid concentration of 20 mg/mL.

### Detergent determination in LacY LUVs

The amount of residual detergent in LacY LUVs was measured by a sugar colorimetric assay (39).

### Efficiency of LacY insertion in LacY LUVs

After protein reconstitution, the final LacY concentration in the LacY LUVs was determined by the biochemical Markwell-Lowry assay (40) using bovine serum albumin as a standard. Proteoliposomes were dissolved with 10 mg/mL sodium deoxycholate and the protein pelleted in 10% (w/v) trichloroacetic acid. The protein precipitate was suspended in 1 mL of alkaline copper reagent (200 mM Na_2_CO_3_, 100 mM NaOH, 7 mM potassium sodium tartrate, 1% w/v SDS, and 0.4% CuSO_4_⋅5H_2_O). 100 *μ*L 50% Folin reagent (v/v) was added and incubated for 1 h before the absorbance was read at 750 nm at room temperature in a Cary 300 UV-Vis spectrophotometer (Agilent Technologies, Santa Clara, CA). The reconstitution efficiency was calculated as a percentage of protein in LacY LUVs compared to the initial amount of LacY added. All sample measurements were performed in triplicate.

### DOPC GUVs preparation by DexPEG hydrogel films and electroformation

DOPC GUVs were prepared by electroformation or DexPEG hydrogel film methods following previously reported methods (35,41), although with lipids sourced from Avanti Polar Lipids. All GUVS were prepared and studied above the main phase transition of the lipids: DOPC (−17°C), DOPE (−16°C), and DOPG (−18°C) for both. Previous work has shown that defects or micron-sized domains can occur in electroformed GUVs that are formed above the main lipid phase transition temperature and then cooled below this phase transition temperature after GUV formation (42). To avoid such defects, GUVs were not subjected to any cooling after formation.

### LacY GUVs preparation by DexPEG hydrogel films

LacY GUVs were prepared and studied above the main phase transition of the constituent lipids based on a previously reconstitution method (16). This method preserves protein activity, as shown for bacteriorhodopsin and Ca^2+^ ATPase, via partial dehydration of protein LUVs with sequential full rehydration under an AC electric field. However, instead of using an electric field to form GUVs, DexPEG hydrogel films were used instead as follows. 20 *μ*L of previously prepared LacY LUVs was transferred to DexPEG hydrogel films in small drops of 2 *μ*L and partially dehydrated under a gentle stream of nitrogen gas for less than 2 min to produce active membrane proteins. A growth chamber was made by placing a polydimethylsiloxane (PDMS) spacer between the LacY LUV-DexPEG hydrogel-coated slide and a microscope glass slide and clamped with crocodile clips (Fig. S2). LacY GUV growth was initiated by hydrating the hybrid LacY LUV-DexPEG hydrogel film with NaPhos (50 mM (pH 7.4)) or phosphate-buffered saline solution (PBS (pH 7.4)) containing sucrose (400 *μ*L, 75 mM sucrose). The hydrated substrates were left to stand overnight at room temperature. Dense suspensions of LacY GUVs were collected the following day from the growth chamber. 10–20 *μ*L of GUVs suspension were diluted in an observation chamber containing 400 *μ*L of glucose buffer (200 mM) for individual microscopy imaging and used for fluctuation analysis.

### LacY GUV preparation by electroformation

LacY LUVs (20 *μ*L) were transferred on an indium tin oxide (ITO)-coated glass slide in small drops of 2 *μ*L and partially dehydrated under a gentle stream of nitrogen gas. A growth chamber was made by placing a PDMS spacer between the LacY LUVs-ITO glass slide and a second ITO glass slide and clamped with crocodile clips. The chamber was filled in with sucrose solution (100 mM), and the electroformation (2.6 V, 10 Hz) was started immediately for 2 h at room temperature, followed by a detachment period (4.4 V, 4 Hz) of 45 min. LacY GUVs were collected, diluted, and immediately used for individual microscopy imaging or fluctuation analysis.

### LacY determination in GUVs by fluorescence confocal microscopy

Quantitative determination of Atto488-LacY in GUVs was performed following the methodology previously published by Marques et al. (43). The angularly averaged fluorescence intensity profiles were extracted with the “radial profile extended plugin” by Philippe Carl from the ImageJ homepage and used to determine the amount of LacY per unit membrane surface and therefore the protein/lipid ratio in GUVs (see details in Figs. S3 and S4).

### Bending rigidity determination in GUVs by fluctuation analysis

The bending rigidity was extracted using a fluctuation analysis technique with the methodology published previously (44,45). The analyzed GUVs presented sizes ranging from 20 to 70 *μ*m and were imaged under phase contrast mode using 1 ms exposure time and recorded for 40 s at 19 ± 1°C. This exposure time is sufficiently short to avoid potential complications due to finite exposure times (15). Fluctuation modes with a wavenumber above ∼1 × 10^−6^ m^−1^ are excluded from the analysis because they fall outside both the spatial and temporal resolution of the measurements reported here. Fluctuation analysis was performed on 4000 contours for each GUV, and the bending rigidity parameter was extracted after fitting the power spectrum in the intermediate regime (modes 6–20). Detailed visual inspection of phase contrast videos showed that GUVs remain spherical with the increase in LacY concentration, but the lipid bilayer appeared to be less stable with a faster loss of the contrast, making contour detection of the lipid bilayer more difficult to detect than with no LacY incorporated.

### Phase contrast and fluorescence confocal microscopy

GUVs were imaged in phase contrast mode on a Nikon Eclipse TE-2000-E inverted microscope (Nikon, Tokyo, Japan) using 20× and 40× objectives, a digital high-speed camera Orca-Flash 4.0 (Hamamatsu, Hamamatsu, Japan), and 100 W TI-DH Dia Pillar Illuminator (Nikon). Epifluorescence imaging was performed on the same microscope using 800 ms exposure time and a mercury light source for providing illumination. Confocal fluorescence microscopy was performed with an AR1 confocal mounted on a Nikon Eclipse Ti-E inverted microscope (Nikon Imaging Centre at King’s College London). Fluorescently labeled LacY was excited with a 488 nm diode laser and detected at the emission wavelength of 525–550 nm.

## Results and discussion

### GUV preparation and LacY reconstitution in GUVs

Pure DOPC GUVs were formed by either electroformation or from DexPEG hydrogel films and their bending rigidity compared. The growth chambers in both preparation methods were built and sealed with PDMS, avoiding the use of silicon grease to eliminate any chance of grease transfer to the final GUV product. The bending rigidity of individual DOPC GUVs was similar between the two GUV preparation methods of electroformation and hydrogel. The bending rigidity of 33 ± 2 *k*_B_T (mean ± standard error (SE), n = 28) obtained by electroformation agrees with that of 30 *k*_B_T previously reported for DOPC GUVs made by an electroformation method (44). DOPC GUVs prepared from hydrogels had a bending rigidity of 37 ± 3 *k*_B_T (mean ± SE, n = 27). The similar values for two preparative methods are consistent with none of the polymer precursors for the DexPEG films being incorporated into the GUVs during their production.

LacY GUVs were prepared from LacY LUVs in a similar way to the partial dehydration and electroformation method (16), but the electroswelling step was replaced by the use of DexPEG hydrogels for GUV generation, which has been proven to be compatible with the use of anionic lipids and physiological ionic strength conditions (35,46) required to preserve biological protein activity in the lipid membrane. For instance, lack of functionality in bacteriorhodopsin was found when the reconstitution was performed with the partial dehydration and electroswelling method just in water (16). Thus, the use of DexPEG hydrogels would assist in the formation of protein GUVs at physiological ionic strength conditions while allowing high GUVs yields. A crucial step to preserve protein functionality relies on a partial dehydration of protein LUVs before rehydration, with full dehydration negatively impacting protein biological activity. Here, LacY LUV partial dehydration was performed on DexPEG hydrogel films instead of ITO conductive surfaces or electrodes, with the presence of the dextran backbone in the hydrogel probably helping to stabilize the protein via hydrogen bonding (47), in a similar manner to trehalose or sucrose used in methods based on full drying and rehydration cycles to form GUVs (48). A single cysteine mutant of LacY, S401C, was fluorescently labeled with the dye Atto488 to allow for fluorescent imaging of the reconstituted protein (see methods in the Supporting materials and methods). Atto488-LacY was reconstituted into the two types of GUVs prepared by DexPEG hydrogel films and electroformation, and the morphology and lipid bilayer quality of the two resulting Atto488-LacY GUVs were compared using phase contrast and epifluorescence microscopy (Fig. S4). Dense suspensions of GUVs were collected from the growth chambers and diluted for individual vesicle microscopy imaging. It has previously been reported that GUVs made via hydrogels give largely defect-free vesicles, whereas GUVs formed by electroformation of charged or neutral lipids can result in morphological changes such as budding and tubulation (49). Our preparations were consistent with this earlier report, with some LacY GUVs with bilayer defects and a nonspherical morphology being observed for GUVs made by electroformation in sucrose solution. Defect-free vesicles prepared by either method looked similar when imaged by either phase contrast microscopy or epifluorescence microscopy, in which the total fluorescence intensity from the Atto488-LacY protein was detected evenly across the surface. GUVs with defects could be readily distinguished and rejected from further analysis. As most of the Atto488-LacY GUVs prepared on hydrogel films with buffer-sucrose solution resulted in spherical morphology with well-defined lipid bilayers and fluorescence, hydrogel films were used to produce GUVs for bending rigidity measurements by fluctuation analysis.

### Lipid dependence of LacY reconstitution

**Figure 1:**
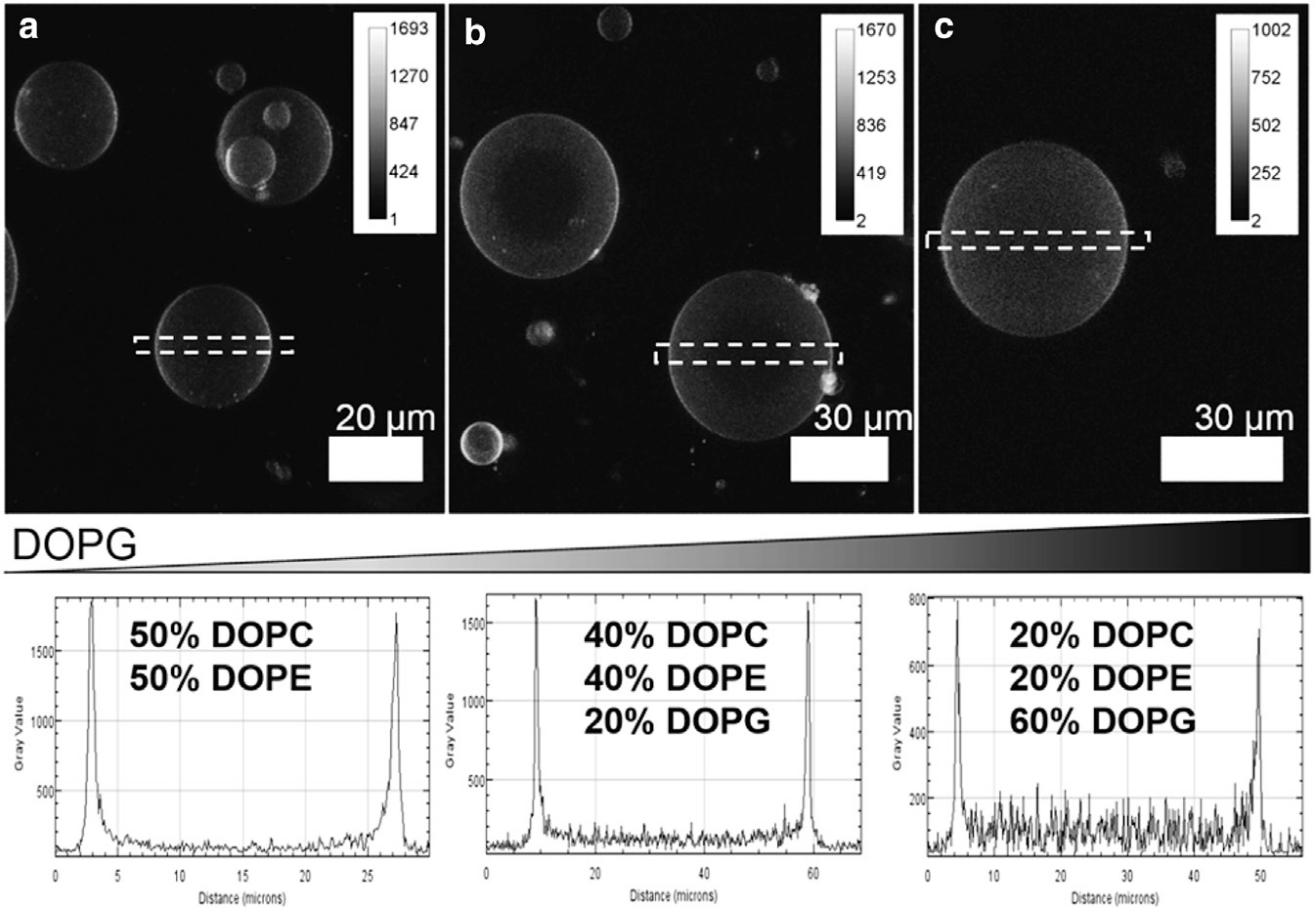
(*Top panels*) Z-projection images from confocal fluorescence microscopy. From left to right are shown Atto488-LacY GUVs as a function of DOPG mol% increase for (*a*) DOPC/DOPE (50:50 mol%), (*b*) DOPC/DOPE/DOPG (40:40:20 mol%), and (*c*) DOPC/DOPE/DOPG (20:20:60 mol%) lipid mixtures. The dotted rectangle in the images indicates the GUV cross section used to determine Atto488 fluorescence intensity across the GUVs shown in the lower graphs. Two intensity maxima (in gray level arbitrary units) in each plot correspond to the fluorescence of Atto488-LacY in the GUV lipid bilayer for one of the confocal slides used for the Z-projection images.

DexPEG hydrogels were interrogated for their ability to produce LacY GUVs using LacY LUVs with lipid compositions DOPC/DOPE (50:50 mol%) and DOPC/DOPE/DOPG (40:40:20 mol% and 20:20:60 mol%), indicating the absence and low and high concentrations of anionic lipid, which directly impact the charge and mechanical properties of the GUV lipid membrane and therefore protein reconstitution and topology. For instance, LacY reconstitution in a LUV DOPC lipid bilayer leads to a modest reconstitution efficiency but low transporter activity. Increasing the amount of DOPE in a DOPC bilayer increases LacY stability and function because of a change in lipid bilayer lateral pressure distribution. On the other hand, correct protein topology is modulated by charge increase in the lipid bilayer with the incorporation of DOPG, but high amounts of DOPG are detrimental to correct topology resulting in the increase of the inverted topology (38,50,51). The presence of LacY in the lipid bilayer of individual GUVs with the lipid compositions described above was validated by confocal fluorescence microscopy using the fluorescently modified Atto488-LacY. The fluorescent protein was reconstituted in LUVs with the three different lipid compositions with a calculated protein/lipid molar ratio of 1:5 × 10^3^. LacY GUVs were produced after the partial dehydration-DexPEG hydrogel procedure. The production of dense suspensions of spherical Atto488-LacY GUVs was observed for all lipid compositions (see Z-projections through the GUV equatorial plane in Fig. 1). Atto488-LacY fluorescence was found to be homogeneously distributed along the lipid bilayer regardless of lipid composition, but the fluorescence intensity in the bilayers decreased as the mol% of DOPG increased (see fluorescence intensity profiles in the *bottom* of Fig. 1). This decrease in the fluorescence intensity is indicative of a lower Atto488-LacY incorporation into the lipid bilayer of GUVs with lipid composition DOPC/DOPE/DOPG (20:20:60 mol%). The results confirm the presence of LacY in GUV lipid membranes. DOPC/DOPE/DOPG lipid mixtures (40:40:20 mol%) were used in experiments to determine the influence of LacY concentration on bending rigidity, as this lipid composition gave good reconstitution yields and is known to support correct LacY topology and function (unlike DOPE/DOPC).

### Reconstitution efficiency and determination of LacY concentration in GUVs

Fluorescence-labeled Atto488-LacY provided a means to determine the concentration of protein in the GUVs. It is important to determine the actual concentration in the final protein GUVs as opposed to merely relying on the initial amount of protein added in LUVs, as the reconstitution is unlikely to be 100% efficient. Besides the relevance of the lipid bilayer composition and lipids charge for protein incorporation in GUVs, the reconstitution of the voltage-gated potassium channel (KvAP) in GUVs showed that the salt concentration is crucial during the rehydration/electroswelling step for the protein incorporation. The formation of KvAP GUVs at physiological salt concentrations yielded GUVs with protein densities higher than those protein densities in the former protein LUVs (52). Here, Atto488-LacY was reconstituted into DOPC/DOPE/DOPG (40:40:20 mol%) LUVs with protein concentrations of 1.3, 2.6, and 5.2 nM corresponding to theoretically calculated protein/lipid molar ratios of 1:1 × 10^4^, 1:5 × 10^3^, and 1:2.5 × 10^3^, respectively. After reconstitution, the effective Atto488-LacY concentration in fluorescent Atto488-LacY LUVs was determined with the Markwell-Lowry assay. These Atto488-LacY concentrations indicated protein insertion efficiencies as follows: 86% (initial protein/lipid ratio 1:1 × 10^4^), 97% (ratio 1:5 × 10^3^), and 56% (ratio 1:2.5 × 10^3^). The insertion efficiency decreased for the protein/lipid ratio 1:2.5 × 10^3^, suggesting protein saturation in Atto488-LacY LUVs. These Atto488-LacY LUVs were used for the formation of Atto488-LacY GUVs with the aid of DexPEG hydrogel films at physiological salt concentrations.

**Figure 2:**
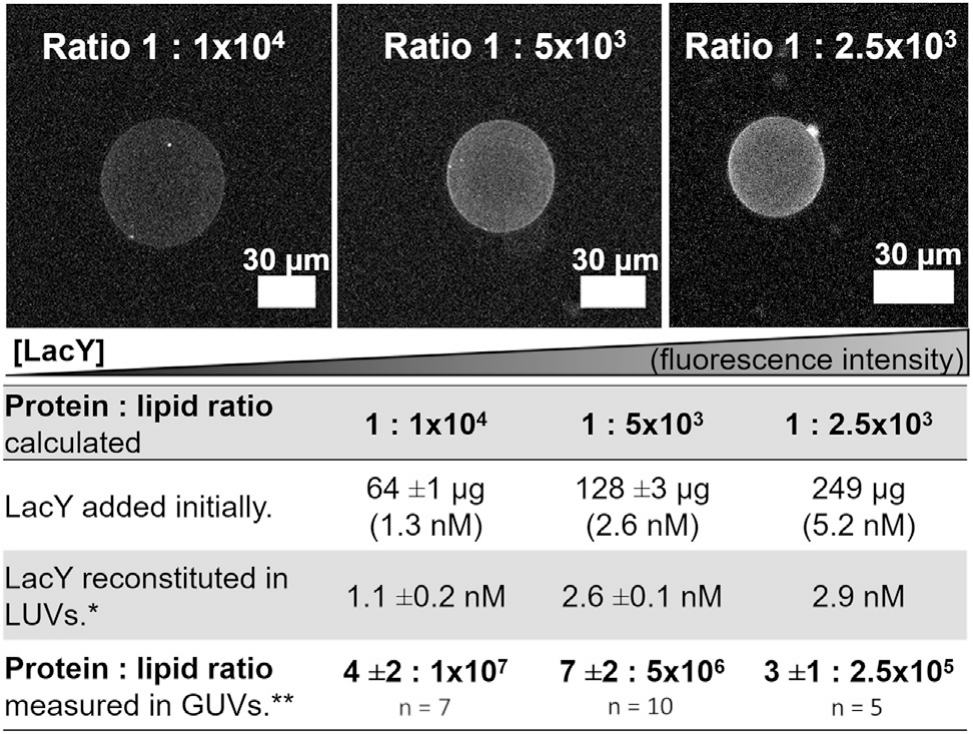
(*Top*) Z-projections of Atto488-LacY GUVs showing protein increase. (*Bottom*) LacY quantification in Atto488-LacY LUVs and Atto488-LacY GUVs with the lipid composition DOPC/DOPE/DOPG (40:40:20 mol%). The protein/lipid ratio given in the first row is that calculated from the initial concentrations used in reconstitution, and the remaining rows are the measured concentrations in the LacY LUVs and LacY GUVs. ^∗^Quantification by the biochemical Markwell-Lowry assay in LUVs. ^∗∗^Quantification by confocal fluorescence microscopy in GUVs. Number of Atto488-LacY ± standard deviation is given. n, number of GUVs measured.

The fluorescence of Atto488-LacY protein was used to quantify the final protein concentration in Atto488-LacY GUVs by confocal microscopy (Figs. S3 and S4). Imaging showed that higher fluorescence intensity was detected in the lipid bilayer of Atto488-LacY GUVs with higher protein concentration (Fig. 2, *top*). The quantitative determination of Atto488-LacY in the lipid bilayer of GUVs showed a dilution of 1000 times from the initially calculated protein/lipid ratios of 1:1 × 10^4^ and 1:5 × 10^3^, whereas 100 times dilution was found for the protein/lipid ratio of 1:2.5 × 10^3^ (Fig. 2, *bottom row* of *table*). The actual protein/lipid ratios determined in GUVs were lower than those predicted from the initial protein concentration used for reconstitution in LUVs because protein is lost during the two-step reconstitution process to produce LacY GUVs.

### Influence of residual detergent on bending rigidity

The effect of residual detergent on the GUV bilayer mechanics was assessed. During protein reconstitution, LUVs are presaturated with an excess of detergents, typically octylphenol polyethylene oxide (Triton X-100) or OG (critical micelle concentration ∼20 mM (53)) to promote protein insertion (16). Therefore, the efficient removal of free detergent in protein LUVs after reconstitution becomes crucial because it might alter the mechanical properties of the GUV lipid bilayer when the detergent presaturated LUVs are transformed to GUVs, directly impacting the bending rigidity interpretation. Commonly, polystyrene Bio-Beads are used for efficient removal of detergent after protein reconstitution (54). GUVs were formed from LUVs, without any LacY present, both in the absence and presence of detergent. LUVs were presaturated with OG 34 mM (1 %wt) in the absence of protein and treated with Bio-Beads to decrease the concentration of OG to 0.34 mM (0.01 %wt). These OG-LUVs with residual detergent were used for GUV production in DexPEG hydrogels and their bending rigidity values determined by fluctuation analysis. The bending rigidity values were compared with a control that corresponds to the formation of GUVs from LUVs without any OG (Table 1). The residual detergent decreased the bending rigidity from 23 to 19 *k*_B_T compared to LUVs without OG preswelling (negative control). Detergent removal with Bio-Beads was optimized by adding an extra step that corresponds to either dialysis or fast dilution of OG preswelled LUVs (55). In both cases, the bending rigidity was similar to the value determined for control GUVs, indicating that adding these treatments to the protocol led to a negligible final effect of any residual detergent on GUVs bending rigidity. Bio-Beads and fast dilution were used for the detergent removal in subsequent experiments because it is a faster method than dialysis.

**Table 1:** Bending rigidities for GUVs with the lipid composition DOPC/DOPE/DOPG as function of the detergent removal method

Detergent removal method	Bending rigidity *k*_B_T	Mean ± SE	GUVs (n)
Negative control	23	±3	13
Bio-Beads	19	±3	18
Bio-Beads and dialysis	21	±2	17
Bio-Beads and dilution	24	±1	34

DOPC/DOPE/DOPG composition is 40:40:20 mol%. The negative control corresponds to bending rigidity value in the absence of OG detergent. Mean ± SE. n, number of averaged individual GUVs.

DDM (cmc ∼0.17 mM) is a commonly used detergent during protein extraction and solubilization of integral membrane proteins because it preserves protein stability and function before protein reconstitution in LUVs, unlike OG, which produces LacY aggregation in solution (56). During protein reconstitution, DDM-stabilized LacY was added to OG-LUVs for protein reconstitution and LacY LUV formation. Therefore, it is important to evaluate the residual DDM and OG detergent mixture after the Bio-Beads and fast dilution method. 0.3 ± 0.2 mM of residual detergent mixture was found after reconstitution of the lowest LacY concentration, which agreed with a control reconstitution in the absence of LacY protein (0.3 mM). Slightly higher residual detergent mixture concentrations were found for reconstitution of higher concentrations of LacY (0.3–0.6 ± 0.1 mM), which is consistent with the faster loss of contrast in the GUV lipid bilayer during fluctuation imaging.

### Influence of LacY and lipid bilayer composition on bending rigidity

**Figure 3:**
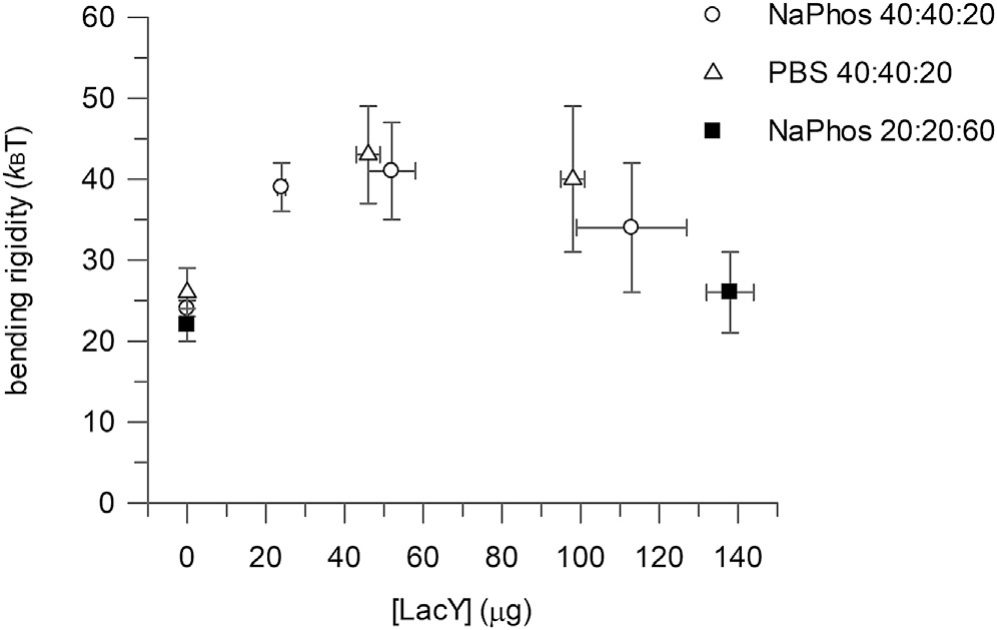
LacY crowding in GUVs with the lipid composition DOPC/DOPE/DOPG (40:40:20 mol%) and (20:20:60 mol%) as a function of the bending rigidity parameter. Bending rigidity values in circles and squares correspond to 50 mM NaPhos and triangles to PBS. Error bar = mean ± SE.

LacY GUVs were used as a minimal cellular membrane model system for evaluating the effect of the protein presence on the mechanics of the lipid bilayer, as determined by bending rigidity. For these bending rigidity studies, LacY without an Atto488 label was used to avoid any influence of this fluorescence label. LacY GUVs were produced in DexPEG hydrogels by the rehydration of previously characterized LacY LUVs with 50 mM NaPhos (low ionic strength (pH 7.4)) or PBS buffers (physiological ionic strength (pH 7.4)). A bilayer composition of 40:40:20 DOPC/DOPE/DOPG (mol%) was used because it is a lipid mix that supports LacY function, with a high percentage of correct structural topology and the ability to transport substrate up a concentration gradient. The presence of the protein in the lipid bilayer increased the membrane bending rigidity (Fig. 3). The bending rigidity of LacY GUVs in both buffers was greater than that for GUVs without any LacY present, regardless of ionic strength; bending rigidity increased from 25 *k*_B_T without LacY present to 43 *k*_B_T (PBS physiological ionic strength) or 41 *k*_B_T (NaPhos low ionic strength) with LacY in the membrane. The optimal hydrophobic match between LacY (hydrophobic length 2.7 nm) and a synthetic lipid bilayer is predicted to be found with a 1,2-dipalmitoleoyl-glycero-phosphocholine bilayer (hydrophobic thickness 2.6 nm) (57), a thinner bilayer than the DOPC (diC18:1) bilayer used here that has an estimated thickness of ∼3.8 nm (with those containing DOPE slightly thicker still (58)). Therefore, the increased bending rigidity is unlikely to be a result of bilayer thickening on the insertion of protein, but rather stiffening due to increased chain lateral pressure. Bending rigidities were also measured in a 20:20:60 DOPC/DOPE/DOPG bilayer. In this lipid composition, LacY function is significantly compromised, with the majority of the protein in the incorrect topology and capable only of supporting downhill transport. Protein-free bilayers in low ionic strength had a bending rigidity of 22 *k*_B_T, similar to the 40:40:20 bilayers, but the addition of LacY had little significant increase in this case, with a bending rigidity of 26 *k*_B_T. The structural rearrangements of the protein itself or in combination with the different lipid properties of the membrane have reduced the effect of protein insertion on bilayer rigidity, at least at the protein/lipid ratios used in this study.

## Conclusion

The use of DexPEG hydrogels provides a viable method to produce robust, artifact-free GUVs for accurate bending rigidity measurements. Moreover, the method enables a broad range of lipid mixtures and buffers to be used. A substantial increase in bending rigidity upon the insertion of LacY was found in LacY GUVs composed of 40:40:20 DOPC/DOPE/DOPG lipids, indicating the stiffening of the lipid bilayer, but there is no further increase detected with greater amounts of the protein in the membrane. In contrast, adding LacY to 20:20:60 liposomes did not significantly change bilayer stiffness. In this study, LacY was used as a model system to investigate the influence of integral membrane proteins on bending rigidity under physiological conditions; we anticipate that the method is transferable to other proteins, allowing for the investigation of the effect of protein size, shape, structure, and concentration, as well as lipid composition, on the physical properties of bilayers with embedded membrane proteins.

## Author contributions

N.L.M., H.E.F., and P.J.B. designed the research. N.L.M. and H.E.F. prepared the experimental samples and conducted the experiments. N.L.M., H.E.F., and P.J.B. analyzed results. N.J.B., S.P., and O.C. wrote the program for fluctuation analysis in GUVs. N.L.M., H.E.F., and P.J.B. wrote the manuscript.

## Supporting citations

Reference (59) can be found in the Supporting material.

## Supporting material

Document S1. Supporting materials and methods and Figs. S1–S5

Document S2. Article plus supporting material
