# Supplementary material for: The membrane transporter lactose permease increases lipid bilayer bending rigidity
Source: Biophys J. 2021 Jul 15;120(17):3787–94. doi: 10.1016/j.bpj.2021.06.038 (PMC8456183; doi:10.1016/j.bpj.2021.06.038)
Supplement: Document S1. Supporting materials and methods and Figs. S1–S5 [file mmc1.pdf]

Biophysical Journal, Volume *120*

Supplemental information

The membrane transporter lactose permease increases lipid bilayer bending rigidity

Nestor Lopez Mora, Heather E. Findlay, Nicholas J. Brooks, Sowmya Purushothaman, Oscar Ces, and Paula J. Booth

SUPPORTING MATERIAL

The membrane transporter lactose permease increases lipid bilayer bending rigidity

Nestor Lopez Mora,[1] Heather E. Findlay,[1] Nicholas J. Brooks,[2] Sowmya Purushothaman,[2,3] Oscar Ces,[2] and Paula J. Booth.[1,*]

[1] Department of Chemistry, Kings College London, Britannia House, 7 Trinity Street, London, SE1 1DB, UK.

[2] Department of Chemistry, Imperial College London, South Kensington Campus, London SW7 2AZ, U.K.

[3] Beyond Meat, 1325 E El Segundo Blvd, El Segundo, CA 90245.

Corresponding Author.

Professor Paula Booth: paula.booth@kcl.ac.uk.

This supporting material includes:

Page	Supporting material
SM-3	**Protein expression and purification.**
SM-3	**Protein labelling.**
SM-4	**Fig. S1**. Absorption spectra of Atto488-LacY.
SM-4	**DexPEG Hydrogel film preparation.**
SM-4	**DOPC GUVs preparation by DexPEG hydrogel swelling.**
SM-5	**DOPC GUVs preparation by electroformation.**
SM-5	**Fig. S2**. Schematic representation for the production of LacY GUVs by DexPEG hydrogel films.
SM-6	**Fig. S3**. Radial intensity profiles and confocal imaging of GUVs dispersed in solutions of the label Atto488 as a function of the concentration.
SM-7	**Fig. S4**. Microscopy calibration curve of fluorescent intensities *versus* concentration of Atto488.
SM-8	**Fig. S5**. Comparison between LacY GUVs prepared by DexPEG hydrogel films and electroformation.
SM-8	**References**.

Protein expression and purification (1, 2). LacY wild type and LacY S401C single cysteine mutant (LacY mutant) of *E. coli* were overexpressed from the pET28a vector in BL21-AI. Cultures were grown in Luria Broth (LB) media at 37 °C to the mid exponential growth phase (OD_{600}=0.8). The cells were induced for 2 hours with 1 mM isopropyl-β-D-1-thiogalactopyranoside (IPTG) or until reaching the growth arrest. Then the cells were harvested by centrifugation, resuspended in PBS supplemented with 10 mM β-mercaptoethanol and protease inhibitor, and storage in the freezer. The cells were cracked by a single passage through a cell disrupter (Constant Systems Ltd) at 25,000 psi. The membranes were collected by centrifugation at 100,000 x g for 30 minutes at 4 °C, resuspended and solubilized for 2 hours at 4 °C in solubilisation buffer containing 50 mM sodium phosphate (NaPhos, pH 7.4), 200 mM sodium chloride (NaCl), 10 mM β-mercaptoethanol, 0.1 mM phenylmethylsulfonyl fluoride (pmsf), 10% glycerol, 2% dodecylmaltoside (DDM), EDTA free protease inhibitor and 20 mM imidazole. The supernatant was cleared by centrifugation for 30 min at100,000 x g, after which the solubilized protein was purified on a Histrap column at 4 °C with the elution buffer containing 50 mM NaPhos, 10 mM β –mercaptoethanol, 0.1mM pmsf, 10% glycerol, 0.05% DDM, 500 mM imidazole. The monomeric LacY was obtained by size exclusion chromatography with the desalting buffer containing 50 mM NaPhos (pH 7.4), 2mM β –mercaptoethanol, 10% glycerol, 0.05% DDM and 0.1 mM pmsf. The purified monomeric LacY was flash frozen and stored at -80 °C until further use in protein labelling or protein reconstitution into LacY LUVs.

Protein labelling. The native serine in the position 401 was replaced for a cysteine (LacY S401C) to allow the conjugation with a fluorescent label through thiol – maleimide chemistry. The previously purified LacY S401C mutant was labelled with Atto488-maleimide reactive dye. Briefly, the monomeric LacY mutant was injected into a Histrap column and incubated for 60 minutes with 1 mM tris (2-carboxyethyl) phosphine (TCEP) in the desalting buffer containing 50 mM NaPhos (pH 7.4), 10% glycerol, 0.05% DDM and 0.1 mM pmsf. The maleimide reactive dye, previously dissolved in the TCEP desalting buffer, was injected into the Histrap column containing the bounded *LacY* S401C mutant in a molar ratio 1 LacY S401C : 30 Atto488-maleimide and incubated for at least 3 hours at 4 °C. Then the excess of free dye was removed by extensive washing with the desalting buffer but this time replacing TCEP for 1 mM β-mercaptoethanol while the LacY mutant is bounded to Histrap column. The resin was drained and washed with elution buffer 50 mM NaPhos (pH 7.4), 500 mM imidazole, 1 mM β-mercaptoethanol, 10% glycerol, 0.05% DDM and 0.1 mM pmsf. Finally, the fluorescent LacY protein was pass through a size exclusion column for desalting and removal of any aggregates formed during the labelling procedure. Protein concentration was measured by absorbance spectroscopy. The purified monomeric fluorescent LacY was flash frozen and storage at -80 °C until further use in protein reconstitution experiments.

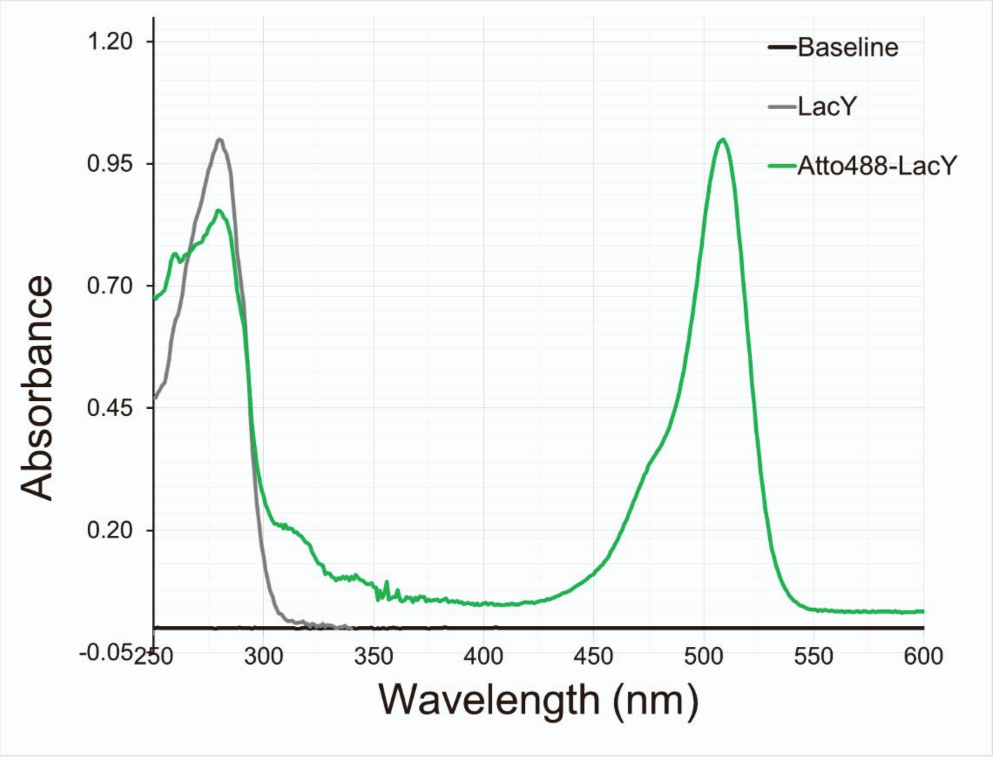

Fig. S1. Absorption spectra of Atto488-LacY.

DexPEG Hydrogel films (3, 4). Maleimide-modified Dextran (1.5 % weight solution) was cross-linked by PEG dithiol at room temperature. Typically for the preparation of 5 glass substrates with DexPEG hydrogel films, Maleimide-modified Dextran (75 mg) (Degree of substitution = 3) was dissolved in water (4.5 g) and 23.6 mg of PEG dithiol (3400 Da) in water (0.5 g) were mixed to provide a hydrogel solution. The mixture was shaken in a vortex for 1 minute and 1 mL of hydrogel solution was immediately drop-casted on thiol functionalised microscope glass slides. The DexPEG substrates were storage at room temperature for their further use.

DOPC GUVs preparation by DexPEG hydrogel swelling. DOPC lipid solution (10 µL, 1 mg/mL in chloroform) was drop-casted on a DexPEG hydrogel coated glass slide. The solvent was evaporated by using a gentle stream of nitrogen gas and keep under vacuum overnight. A swelling chamber was made by placing a polydimethylsiloxane (PDMS) spacer between the hybrid lipid-DexPEG hydrogel coated slide and a microscope glass slide and clamped with crocodile clips. GUV growth was initiated by hydrating the lipid-DexPEG hydrogel film with 400 µL of filtered sucrose solution or sucrose buffer (450 mM). The hydrated hybrid films were left to stand overnight at room temperature. Dense suspensions of GUVs were collected from the growth chamber the following day and used immediately.

DOPC GUVs preparation by electroformation. DOPC GUVs were prepared by the electroformation method (5). DOPC lipids (10 µL, 1 mg/mL in chloroform) were drop casted on an indium tin oxide (ITO) microscope slide. The ITO microscope slide with lipids was place under a gentle stream of nitrogen gas for the evaporation of the solvent and keep under vacuum overnight. An electroformation chamber was made by placing a PDMS spacer between the ITO and a microscope glass slide and clamped with crocodile clips. A filtered sucrose solution (400 µL, 450 mM) was injected to the electroformation chamber. GUV growth was initiated by applying an alternating voltage (2.6 V, 10 Hz) for 2 hours followed by a detachment step of 45 minutes (4.4 V, 4 Hz). GUVs were collected from the chamber and used immediately.

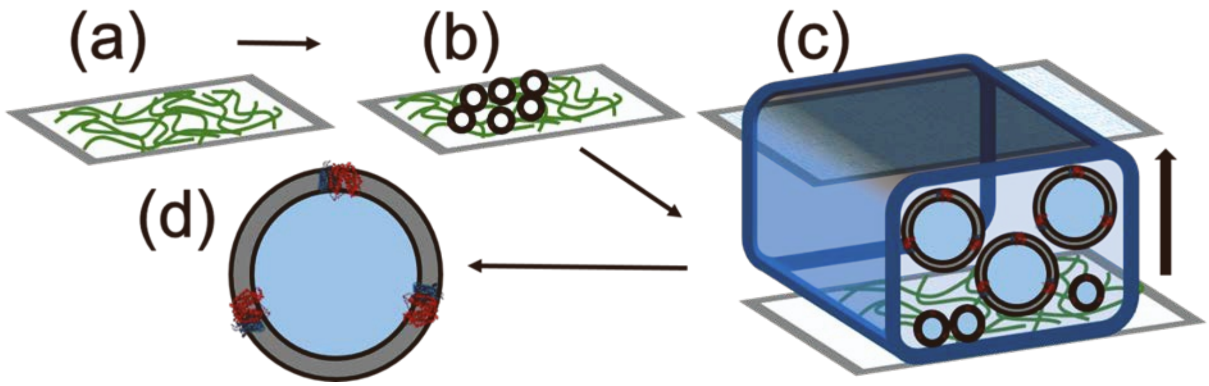

Fig. S2. Schematic representation for the production of LacY GUVs by DexPEG hydrogel films. (a) DexPEG hydrogel substrate. (b) Deposition of LacY LUVs on the surface of DexPEG hydrogel films. (c) Schematic representation of the growth chamber, rehydration and LacY GUVs growth. (d) Illustration of one LacY GUV for microscopy imaging.

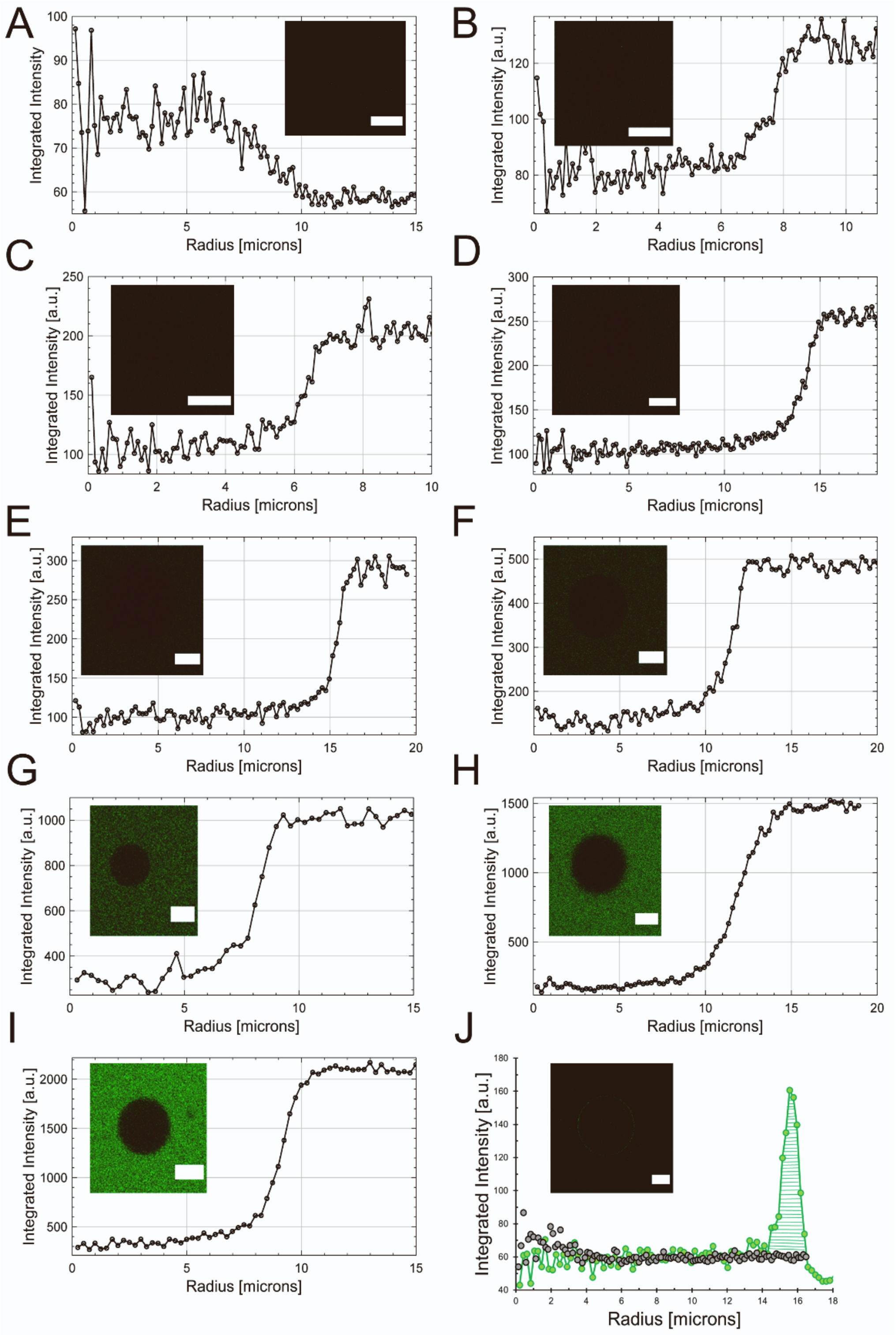

Fig. S3. Radial intensity profiles and confocal imaging of GUVs dispersed in solutions of the label Atto488 as a function of the concentration. A) 0 mM Atto488, B) 4 mM Atto488, C) 6 mM Atto488, D) 8 mM Atto488, E) 10 mM Atto488, F) 20 mM Atto488, G) 40 mM Atto488, H) 60 mM Atto488, I) 80 mM Atto488 and J) Typical confocal imaging of Atto488-LacY GUVs and

radial intensity profile for the calculation of the number of LacY per unit membrane surface (Γ) extracted from Equation 1. The green area below the peak is proportional to Γ. Scale bars are 10 µm.

$$\Gamma = \frac{C}{R} \int_0^\infty I(r) - I_0(r)\, rdr \quad \text{Equation 1}$$

where *I(r)* is the radial profile intensity of Atto488-LacY in the GUV membrane, $I_0(r)$ the intensity profile in the absence of fluorescent label, *R* the radius of the GUV and *C* the concentration determined from Fig. S4. The protein : lipid ratio was determined with the number of Atto488-LacY per $1x10^7$, $5x10^6$ and $2.5x10^6$ lipids (N_L) by using Equation 2.

$$N_{Atto488-LacY} = N_L\, \Gamma\, A_L \quad \text{Equation 2}$$

where A_L is the area for one DOPC of 0.7 nm^2.

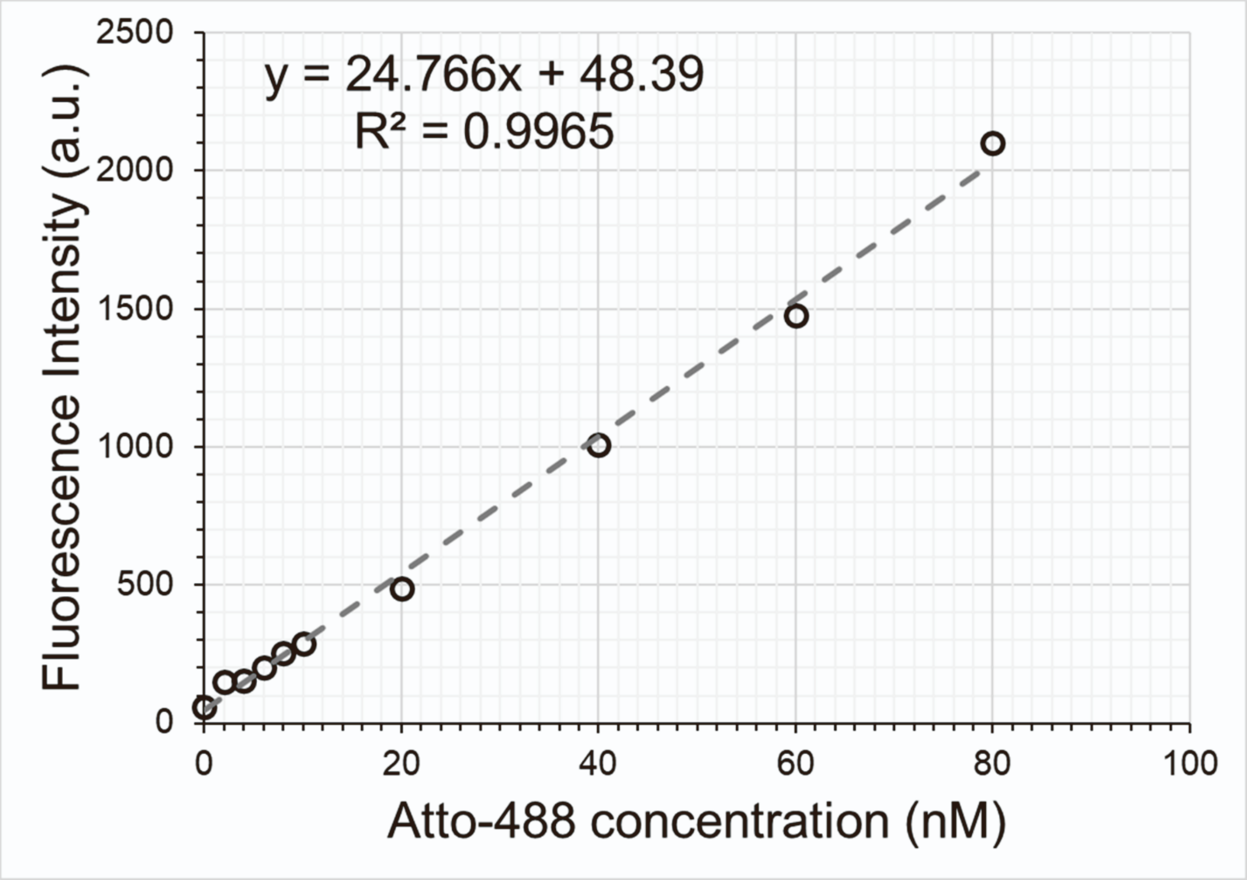

Fig. S4. Microscopy calibration curve of fluorescent intensities versus concentration of Atto488. The fluorescence intensity of Atto488 tag alone (Fig. S3 A-I) was found to be linear with increase in concentration. This linear dependence was used to calibrate the fluorescence of Atto488-LacY GUVs by correlating the fluorescence intensity in the lipid bilayer of Atto488-LacY to the fluorescence intensity of the label Atto488 as a function of the concentration to determine *C*.

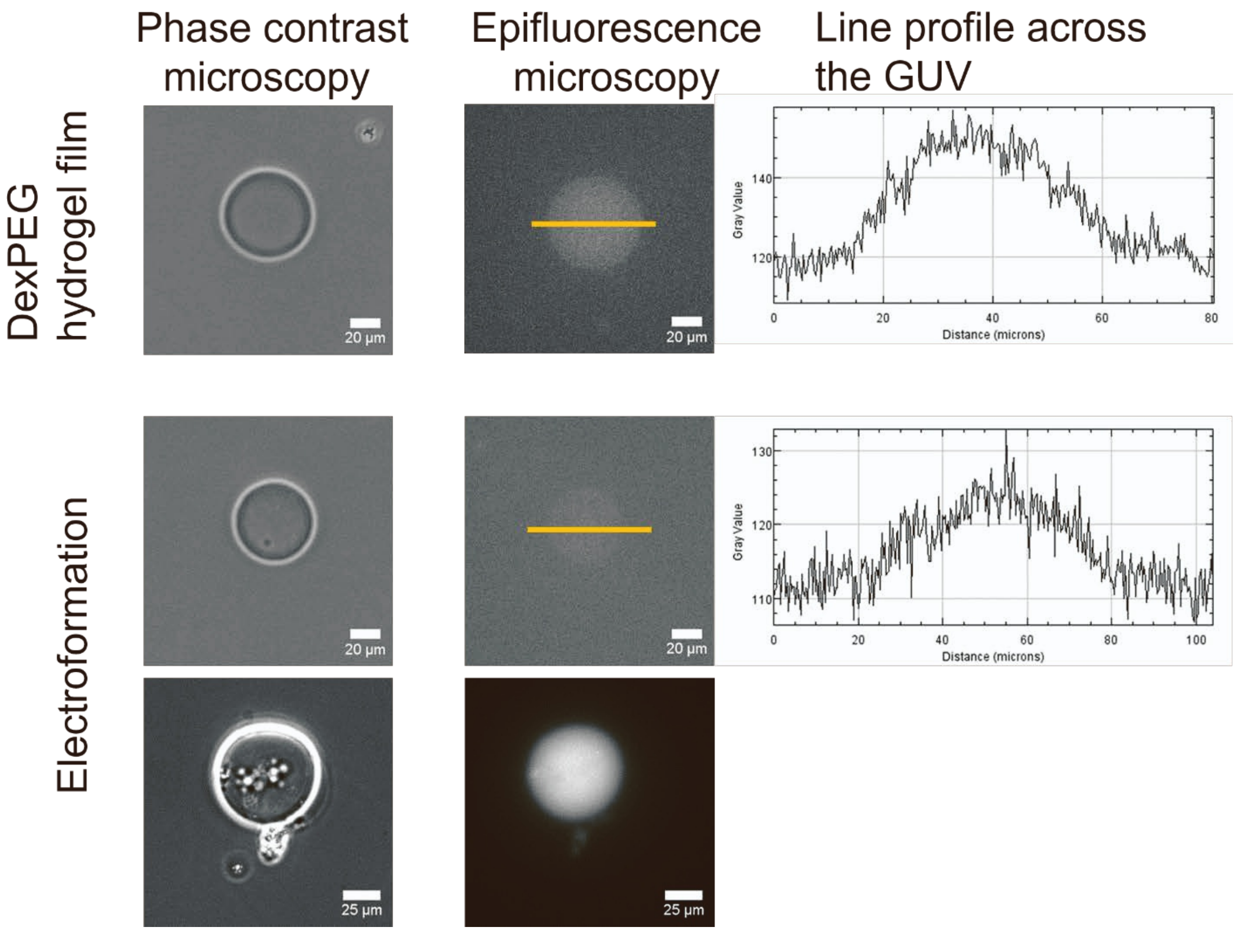

Fig. S5. Comparison between Atto488-LacY GUVs prepared by DexPEG hydrogel films and electroformation. A mutant LacY with a single cysteine in a cytoplasmic loop, S401C, was fluorescently labelled with the dye Atto488 via thiol-maleimide chemistry to yield Atto488-LacY (see details in the protein labelling section), so that protein inserted into the bilayer could be visualised by fluorescence. Atto488-LacY was reconstituted into the GUV lipid bilayer using the DexPEG hydrogel and the electroformation methods. GUV imaging was performed using bright field and epifluorescence modes. Micrographs on the left correspond to phase contrast microscopy and micrographs on the right to epifluorescence microscopy across the surface of the GUV, with Atto488-LacY incorporated in the lipid bilayer. The intensity profile in grey levels (a.u.) across the GUV is indicated with a yellow line in the epifluorescence micrographs. GUVs with defects can be seen under phase contrast and often show brighter and/or uneven fluorescence due to internal structures and possible protein aggregation

Supporting References

1. Harris, N. J., H. E. Findlay, J. Simms, X. Liu, and P. J. Booth. 2014. Relative domain folding and stability of a membrane transport protein. J Mol Biol 426:1812-1825.

2. Findlay, H. E., N. J. Harris, and P. J. Booth. 2016. In vitro synthesis of a Major Facilitator Transporter for specific active transport across Droplet Interface Bilayers. Scientific Reports 6.
3. Mora, N. L., J. S. Hansen, Y. Gao, A. A. Ronald, R. Kieltyka, N. Malmstadt, and A. Kros. 2014. Preparation of size tunable giant vesicles from cross-linked dextran(ethylene glycol) hydrogels. Chem Commun 50:1953-1955.
4. Mora, N. L., Y. Gao, M. G. Gutierrez, J. Peruzzi, I. Bakker, R. J. R. W. Peters, B. Siewert, S. Bonnet, R. E. Kieltyka, J. C. M. van Hest, N. Malmstadt, and A. Kros. 2017. Evaluation of dextran(ethylene glycol) hydrogel films for giant unilamellar lipid vesicle production and their application for the encapsulation of polymersomes. Soft Matter 13:5580-5588.
5. Angelova, M. I., and D. S. Dimitrov. 1986. Liposome electroformation. Faraday Discussions of the Chemical Society 81:303-311.
